# The Uptake and Vaccination Willingness of COVID-19 Vaccine among Chinese Residents: Web-Based Online Cross-Sectional Study

**DOI:** 10.3390/vaccines10010090

**Published:** 2022-01-08

**Authors:** Yi Kong, Hao Jiang, Zhisheng Liu, Yi Guo, Dehua Hu

**Affiliations:** 1 Department of Biomedical Information, School of Life Sciences, Central South University, Changsha 410013, China; kongyi@csu.edu.cn (Y.K.); jianghao1209@csu.edu.cn (H.J.); 220126@csu.edu.cn (Z.L.); 2 Xiangya School of Medicine, Central South University, Changsha 410013, China

**Keywords:** COVID-19 vaccine, vaccination willingness, vaccine hesitancy, influential factor

## Abstract

Objective: To investigate the uptake and vaccination willingness of the COVID-19 vaccine among Chinese residents and analyze the difference and factors that impact vaccination. Methods: The snowball sampling method was used to distribute online questionnaires. Relevant sociodemographic data along with the circumstances of COVID-19 vaccination were collected from the respondents. The χ^2^ test, independent samples *t* test and binary logistic regression analysis were used to analyze the data. Results: Among 786 respondents, 84.22% had been vaccinated. Over 80% of the vaccinated population have completed all the injections because of supporting the national vaccination policies of China, while the unvaccinated population (23.91%) is mainly due to personal health status. Meanwhile, statistical analysis revealed that the main predictors of not being vaccinated were younger age (3 to 18 years old), personal health status, and lower vaccinated proportion of family members and close friends (*p* < 0.05). Conclusions: There was a high level of uptake of the COVID-19 vaccine in China, and people who have not been vaccinated generally had a low willingness to vaccinate in the future. Based on our results, it suggested the next work to expand the coverage of the COVID-19 vaccination should be concentrated on targeted publicity and education for people who have not been vaccinated.

## 1. Introduction

COVID-19 (SARS-CoV-2) ravaged the world severely from December 2019, with more than 281 million confirmed cases and more than 5.4 million deaths as of 29 December 2021 [1]. Vaccination has been proved to be the most economical and effective means to control infectious diseases, and the establishment of herd immunity through large-scale vaccination has been regarded as an effective and essential way to block the transmission of the pandemic [2,3]. In China, an emergency use program was launched to vaccinate a limited number of healthcare workers and other occupations at high risk of infection in July 2020 [4]. At the end of 2020, China officially approved the launch of COVID-19 vaccines and provided them to the whole population free of charge. According to Guidelines of vaccination for COVID-19 vaccines in China (First edition) [5], three types of vaccines were approved in the market for general use in China in March 2021, including the SARS-CoV-2 inactivated vaccine (Vero cells) produced by the Wuhan Institute of Biological Products and Wuhan Institute of Virology and Sinovac Biotech Co., Ltd., the recombinant adenovirus type-5-vectored COVID-19 vaccine produced by Tianjin Cansino Biotechnology Inc., and the SARS-CoV-2 subunit vaccine produced by Anhui Zhifei Longcom Biopharmaceutical Co., Ltd. Then vaccines were provided to transmission risk populations, such as people who were at risk of occupational exposure to coronavirus and study or work abroad [4]. Nationwide vaccinations were then carried out. As of 31 December 2021, a total of 2.84 billion doses of vaccines had been reported in 31 provinces (autonomous regions and municipalities directly under the central government) and Xinjiang Production and Construction Corps in China [6].

Before the vaccines were conditionally approved, some studies were conducted on the willingness to receive COVID-19 vaccines. A global survey showed that participants from China gave the highest vaccination willingness rate (88.60%) while participants from the other 18 countries gave the proportion from 54.85% to 85.63% in June 2020 [7]. Meanwhile, a nationwide investigation also found that the free vaccination policy played an important role in improving the vaccination willingness among Chinese residents during the same period [8]. Other studies were conducted in the early period of the launch of the COVID-19 vaccine policy in the first half-year of 2021, and the willingness of the residents was relatively higher than in previous studies [9,10]. However, studies have also shown the real vaccination rate of other pandemic vaccines could be much lower than the acceptance after the introduction of the vaccine and promotion of mass immunization programs [11]. Those who have not been vaccinated against COVID-19, or who have been vaccinated but have not completed all the injections may hinder the comprehensive promotion of the vaccination and reduce the possibility of receiving the booster injection in the future.

Compared with other pandemics of influenza, the COVID-19 pandemic has shown higher severity in terms of transmissibility, mortality, length of duration and variability of the COVID virus. A study has shown the coverage, efficacy and speed were all of significance for the COVID-19 vaccine response [12], which points out that countries around the world may need to make a greater effort in vaccination policies to control the pandemic and resume normal living.

In this case, understanding the current coverage of vaccination and identifying common barriers and facilitators of the uptake of the COVID-19 vaccine are important to predict the maximum vaccination rate, expand the coverage of vaccination and form the herd immunity efficiently. There are few studies on both the vaccine uptake among the general population and the willingness to uptake the COVID-19 vaccine among unvaccinated individuals in China at present. This study aims to evaluate the coverage of the vaccination, the possibility of unvaccinated individuals receiving vaccination in the future, as well as the influencing factors of the reception of the COVID-19 vaccine among the Chinese population. This information is critical to making accurate countermeasures to increase the vaccination rate to strengthen herd immunity.

## 2. Materials and Methods

### 2.1. Study Design and Participants

This study was designed to explore the determinants of the uptake of the COVID-19 vaccine among Chinese residents and the future vaccination intentions of unvaccinated residents. Ethical approval to conduct the study was obtained from the Institutional Review Board of College of Life Sciences, Central South University (Reference No.:2021–1–41) and conducted following the guidelines of the Declaration of Helsinki.

On the basis of literature research and expert consultation, all research members initially designed a questionnaire consisting of 50 questions, including sociodemographic data, vaccination details, and latent influencing factors. To make the content of the questionnaire more credible and the structure more reasonable, we conducted an in-depth interview, and the outline of the interview was designed based on the questionnaire. The whole in-depth interview was conducted for a total of five days from 19 June 2021 to 23 June 2021, 16 interviewees were invited with different characteristics of gender, age, living area, educational background, and occupation. The entire interview content was recorded with the interviewee’s permission, the interviewer remained neutral at all times during the interviews. Based on the opinions collected from interviewees, we optimized our questionnaire by combining similar questions and making the content of questions more complete, such as the factor of the COVID-19 vaccine’s price being eliminated according to the implementation of the free vaccination policy, the range of reasons for reception and refusal of vaccines were expanded and the sources of vaccine information were supplemented.

The formal questionnaire is confirmed in a non-scale form based on the results of literature research, expert advice, and in-depth interview. There were 34 questions in the questionnaire, covering the following data: participants’ sociodemographic data (like gender, age, living area, educational background and occupation), participants’ COVID-19 vaccination details (like the brand of the vaccine, the organizer of the vaccination, the progress of the vaccination and the possibility to receive future injections (for those who had not finished all injections or had not received any injections)), and participants’ attitudes towards the COVID-19 pandemic and vaccines (the score ranges from 1 to 5 points, the more attention that was paid to the COVID-19 pandemic and the higher the trust in vaccines, the higher the score). Besides, the proportion of the vaccination of participants’ family members and close friends, the amount and sources of COVID-19 vaccines’ information were assessed in the questionnaire.

During the national promotion and implementation of the COVID-19 vaccination policy, this cross-sectional online study was carried out from 24 July 2021 to 7 August 2021, for a total of two weeks. Compiled through the software of “Wen Juan Wang” (https://www.wenjuan.com/, accessed on 16 September 2021), the questionnaire was mainly distributed in the form of web links and QR codes via social software such as WeChat, QQ and Weibo. To prevent repeated answers, an IP address was limited to submitting answers only once. Using the snowball sampling method, participants were first recruited randomly online and requested to spread other questionnaires to other eligible participants. Inclusion criteria were: (1) living in China mainland for more than six months, (2) informed consent and voluntary participation in this study. Exclusion criteria were: (1) the time spent on answering the questionnaire is less than one minute, (2) choosing gender as male while answering the questions restricted to female, (3) refusing to participate in this study.

### 2.2. Statistical Analysis

After exporting the data of the survey, the software of Excel 2020 was used to establish a database, and the original data was screened and adjusted. Participants’ characteristics were summarized using frequencies and percentages. Univariate analysis was used to examine descriptive information for each variable. Separate bivariate analyses were applied to examine the correlation of each variable with the reception of the COVID-19 vaccination. Crosstabs were generated for categorical variables, and the independent sample *t* test was used for two continuous variables, the scores of the attitudes towards both the COVID-19 pandemic and vaccines. Lastly, we used binary logistic regression analyses on all variables to analyze the influencing factors of the reception of COVID-19 vaccination and selected our final model using a backward procedure. The statistical significance was defined as *p* < 0.05 and all statistical analyses were performed using SPSS 25.0. (IBM Corp., IBM Corp, USA, 2017).

## 3. Results

### 3.1. Participant Characteristics

The study included 786 respondents, 347 (44.15%) were male and 439 (55.85%) were female. Of the participants, 78.5% of them were 18–35 years old and the median age was 28.83 years old. Most of them were urban residents 678 (86.26%) and highly educated with a college degree or above (74.55%). Their occupations were mainly employees of enterprises and institutions (34.10%) and students (27.86%); 662 respondents (84.22%) had received COVID-19 vaccination. The exact numbers and characteristics of the respondents are presented in Table 1.

### 3.2. Characteristics of Vaccinated and Unvaccinated Participants

Among vaccinated participants, 295 (44.56) were male and 367 (55.44) were female. The vast majority of them were from 18 to 35 years old (80.06%), living in urban areas (85.65%), with a college degree or above (75.68%). Most of them were employees of enterprises and institutions (34.10%) and students (27.86%) (Table 1).

Further, 662 participants had received the COVID-19 vaccination, and more than 80% of them have completed the whole primary vaccination course. The main reason for vaccination was to support the national vaccination policies (48.10%) according to the analysis of the multi-choice question. Among the five brands of COVID-19 vaccines in China, the vaccines produced by Beijing Sinovac Biotech Company accounted for the highest proportion (50.6%). Of these, 281 people (42.45%) were vaccinated in their community. Reports from the official online media (34.98%) and the authoritative news media (28.67%) were the main channels for participants to obtain information on the COVID-19 vaccine. (Table 2).

There were 112 unvaccinated participants in this study; of these, 58.06% were female. 81.45% were in the age group being between 18 and 35 years in age. In terms of living area, 89.52% were urban residents. College degree accounted for the highest education background group (40.32%), followed by master’s degree and above (28.23%), high school and below (20.97) and associate college (10.48%). 43.55% of them were employees of enterprises and institutions. (Table 1).

According to the results of the answers to the multiple-choice question in Table 3, 124 participants were unvaccinated because of the following reasons: personal health status (23.91%), worried about side effects or adverse reactions (20.11%) and the belief in the safety of living area (14.13%). Without considering the price, only 33.07% of the unvaccinated participants had relevantly high willingness about COVID-19 vaccine, indicating that most of them may not get vaccinated in the future. About 35% of the participants had no preference for the type of vaccine, and nearly 50% preferred to be vaccinated in the community, which was consistent with vaccinated participants’ choice. Reports from official online media and authoritative news media were the main sources of COVID-19 vaccine information, accounting for 32.27% and 28.72%, respectively.

### 3.3. Influencing Factors of the Uptake of COVID-19 Vaccines

The result of the questionnaire showed that there were still 15.78% of participants who had not received COVID-19 vaccination. The comparison of baseline characteristics between the two groups with the χ^2^ test and an independent sample *t* test was displayed in Table 3. The statistical analysis indicated that health status, attitudes towards COVID-19 pandemic, attitudes towards COVID-19 vaccines and the proportion of family members and close friends’ vaccination were significantly related to Chinese residents’ vaccination refusal. (Table 4).

The results of binary logistic regression, including significant factors at the 5% level of χ^2^ test and independent-sample *t* test, suggested that those younger than 18 years of age (*p* < 0.05), those who hesitated to get vaccinated due to personal health status (*p* < 0.001), and those who had a lower portion of vaccinated close friends and family members (*p* < 0.001) were less likely to get vaccinated against COVID-19. These revealed that the main predictors of not being vaccinated were the age (3 to 18 years old), personal health status, and lower vaccinated proportion of family members and close friends (Table 5).

## 4. Discussion

There are 786 participants in this survey, 662 (84.22%) have received COVID-19 vaccination, which proves that the actual vaccination rate of Chinese residents is higher than the previous survey results of vaccination willingness [8,9,13]. In addition, 553 (70.63%) have completed the whole primary vaccination course as of 7 August 2021 and have just met the minimum requirement of vaccination rate for the formation of herd immunity under the premise of optimal effectiveness of the COVID-19 vaccine [3,14]. However, the proportion of the whole country who completed the entire vaccination meets the herd immunity requirement as of 16 September 2021 [15], which is five weeks later than our results. This may be closely related to the fact that most of the participants in our research were in line with the age requirements (from 18 to 59 years old) of vaccination at that time, and we have not been able to conduct a thorough investigation among all age groups. Meanwhile, people from this age group have greater needs in work and interpersonal communication and are more likely to be vaccinated to immunize themselves from infection.

Other studies have shown that women, young people, people living in urban areas, and people with higher education levels were more hesitant to get vaccinated and concerned about the safety and effectiveness of the vaccine [11,16,17,18]. Relating to the free vaccination policy [8] and its thorough implementation, these differences above are not reflected in this study. In general, residents in China have a strong sense of epidemic prevention after an arduous fight against COVID-19. Many of them are determined to accept vaccinations for the reason of promoting the COVID-19 vaccine policy. Thus, there are no significant differences in vaccine hesitation among the various characteristics of participants. However, it should be noted that some people (10%) received the vaccine without knowing the manufacturer of the vaccine. To a certain extent, it reflects the public’s trust in the safety of China’s COVID-19 vaccines while it manifests that quite a few people lack knowledge of COVID-19 vaccines and have no awareness of their vaccination needs. They may merely take vaccination out of conformity psychology, which illustrates the publicity of vaccine knowledge still needs to be further strengthened.

Binary logistic regression analysis shows that the vaccination rate of those younger than 18 years old (*p* < 0.05) is significantly lower than those of other age groups, which is directly related to the fact that COVID-19 vaccination among minor groups is not fully available in China during this period. The vaccination of minor groups against COVID-19 is an important step in the process of establishing herd immunity for the whole population. The government has gradually allowed the vaccination of 3–17 years old, especially 12–17 years old, from June to August 2021 [19,20]. Additionally, whether the group of minors can achieve vaccination is firmly related to the intention of parents or guardians. Multiple survey results have shown that the degree of hesitation of parents to vaccinate their children against COVID-19 varies from country to country but is higher than that of parents’ hesitation to vaccinate themselves. Concerns about vaccine safety and effectiveness are common factors that reduce parents’ willingness to vaccinate their children [21,22,23,24]. A feasible method is that authorities should release authoritative information on the safety and reliability of vaccines in time and reinforce publicity and education for parents through schools and communities. Organizational work of vaccination in schools is essential to improve the convenience of vaccination for young people. Providers of vaccination services may strengthen the education about vaccination procedures, vaccination contraindications, vaccination of children with special health status, and the treatment process of adverse reactions, so as to improve their professionalism. Furthermore, advice from doctors may also help to reduce and eliminate parents’ doubts about the COVID-19 vaccines [24].

Besides, people who hesitate to receive the vaccine due to personal health status have a lower willingness to receive the vaccine (*p* < 0.05). This is consistent with the results of previous studies. For example, people with underlying diseases were generally less willing to be vaccinated [25,26,27,28,29,30,31,32] as they were more likely to refuse to be vaccinated due to worrying that the vaccination would aggravate their underlying diseases. This was more likely for women who were pregnant or preparing for pregnancy to refuse vaccinations, studies showed that they may hesitate to uptake the vaccine [33,34] due to the concerns about vaccine safety that large sample studies on the safety of vaccines in this group were invalid [33,35]. It can be predicted that there will always be a certain unvaccinated population, including but not limited to those who could not be vaccinated due to personal health status. Therefore, public health institutions should carry out targeted publicity and education consistently to lower the possibility of infection even if they are not protected by the COVID-19 vaccine, and self-protection measures spread via official online media and professional advice from community doctors may help [24]. In-depth vaccine research and development on large samples of specific sub-groups should be continued to further improve and verify the safety and effectiveness of vaccines, so as to make it possible for them to get vaccinated against COVID-19 as soon as possible.

As with previous research, a lower proportion of vaccinated family members and close friends was associated with a lower probability of individuals’ vaccination (*p* < 0.05) [18,36,37,38,39,40]. A survey formed on the Health Belief Model was conducted in January 2021, indicating that perceived barriers and recommendations from friends or family were also associated with vaccination behavior directly and in combination, since family and friends may affect individuals’ vaccination willingness or behavior through daily communication. In this regard, communities are advised to organize collective vaccination in various forms, such as families or work units to encourage more eligible people to participate in vaccination and increase the coverage rate of vaccination. Moreover, public health institutions may improve the publicization work from an altruistic motivation perspective, which emphasizes that people can protect not only themselves but also their loved ones by being vaccinated [18].

Compared with social media, participants in this survey tend to pay more attention to reports from the official online media and authoritative news in the news media, to some extent which reduces the possibility of widespread dissemination of false vaccine information. This is inconsistent with the results of previous studies. In general, the internet played a role in promoting anti-vaccine campaigns [41], and various media, especially social media, were essential in shaping vaccine-related public opinion [16,42], while a large number of false information disseminated on the internet would make people have wrong and negative views on vaccines and deter them from vaccination [16,43,44,45,46]. The results of this study are related to the fact that China has attached great importance to the prevention and control of COVID-19 and vaccine publicity, and that people tend to pay attention to the information provided by authoritative information channels in order to obtain effective vaccine information in a timely manner. On the basis of public confidence, government and public health institutions should maintain the authenticity and timeliness of released information, which may provide a reference for other countries in the management of vaccines or other health information.

It is worth noting that unvaccinated participants have a low willingness to vaccinate in the future, which is closely related to the acceptance delay or even refusal of vaccination. In this respect, increasing the public’s willingness is key to expanding the vaccination coverage in China. A study demonstrates that scarcity of vaccines reduced participants’ vaccination intentions, so it is essential to ensure an adequate supply of vaccines to increase demands for vaccines [47]. Propagation of scientific knowledge related to the pandemic and vaccines should be further implemented on official online media [48], such as sharing the findings of COVID-19 vaccine-related studies (including safety and effectiveness of vaccines) and vaccine’s approval process [42], dealing with negative responses to vaccines and safety issues [24]. Moreover, experts (such as doctors) and political figure endorsements may boost the public’s vaccination willingness by popular science lectures on COVID-19 vaccines [24,48,49]. In particular, taking advantage of an educational video demonstrated a significant impact on the acceptance of vaccines in older people. [50].

## 5. Conclusions

This study reflects that there exists a high coverage of the COVID-19 vaccination and a low willingness of unvaccinated populations by June 2021 in a time of increasing vaccine supply in China.

The main conclusions are as follows:

(1) The vaccination rate in China is higher than the willingness showed in a series of previous surveys. Owing to the implementation of a free vaccination policy and supporting the work of public health institutions, Chinese residents are vaccinated to immunise both themselves and others from the coronavirus.
(2) Family members and close friends have an impact on the uptake and vaccination willingness of the COVID-19 vaccine among Chinese residents. Thus, collective vaccination and publicization work emphasized the value of helping others by receiving injections are essential to expanding the vaccine coverage.
(3) Publicity and education about vaccines need to be further promoted in China. Public health institutions and medical workers play an important role in making it clearer for residents to understand the knowledge of vaccines, especially the safety and effectiveness, to increase the willingness of vaccination.
(4) Online publicity methods are of significance to release the vaccine information, especially the official online media. Research and development of vaccines and the national vaccination progress should be updated to the public at a certain frequency.

## 6. Limitations and Prospects

This study still has several limitations. First of all, the questionnaire is distributed via the internet, which may impose restrictions on the representativeness of the present study’s sample. Secondly, the participants of the survey filled out the questionnaire anonymously and the authenticity could not be verified. Thirdly, this study is a cross-sectional study, and it is hard to follow up the future behaviors of those who have not completed all the injections and have not been vaccinated.

Further studies are needed to focus on comparing the similarities and differences of the residents’ vaccination in different periods of the COVID-19 pandemic. On account of the necessity of herd immunity, more attention should be paid to the uptake of unvaccinated populations, such as people under 18 years old and with lower physical fitness levels. Moreover, whether people are vaccinated as scheduled and choose to receive booster injections are also of importance in this field. Conducting follow-up surveys and utilizing the data from public health institutions may help to ensure the accuracy of the results.

## Figures and Tables

**Table 1:** Sociodemographic characteristics of survey participants (*n* = 786).

Characteristic	Contents	Participants, *n* (%)	Vaccinated Participants, *n* (%)	Unvaccinated Participants, *n* (%)
Gender	Male	347 (44.15)	295 (44.56)	52 (41.94)
	Female	439 (55.85)	367 (55.44)	72 (58.06)
Age group	Under 18	21 (2.67)	6 (0.91)	15 (12.10)
	18–35	617 (78.50)	530 (80.06)	87 (70.16)
	36–59	115 (14.63)	101 (15.26)	14 (11.29)
	60 and above	33 (4.20)	25 (3.78)	8 (6.45)
Living area	Urban	678 (86.26)	567 (85.65)	111 (89.52)
	Rural	108 (13.74)	95 (14.35)	13 (10.48)
Educational background	High school and below	107 (13.61)	81 (12.24)	26 (20.97)
	Associate college	93 (11.83)	80 (12.08)	13 (10.48)
	College degree	364 (46.31)	314 (47.43)	50 (40.32)
	Master’s degree and above	222 (28.24)	187 (28.25)	35 (28.23)
Occupation	Employees of enterprises and institutions	268 (34.10)	214 (32.33)	54 (43.55)
	Medical workers	60 (7.63)	58 (8.76)	2 (1.61)
	Civil servants	101 (12.85)	89 (13.44)	12 (9.68)
	Teachers	49 (6.23)	43 (6.50)	6 (4.84)
	Students	219 (27.86)	184 (27.79)	35 (28.23)
	Farmers	24 (3.05)	22 (3.32)	2 (1.61)
	Retired	41 (5.22)	32 (4.83)	9 (7.26)
	Others	24 (3.05)	20 (3.02)	4 (3.23)
Uptake of the COVID-19 vaccine	Yes	662 (84.22)		
	No	124 (15.78)		

**Table 2:** Characteristics of Vaccinated Participants (*n* = 662).

Variables		Participants, *n* (%)
Reasons for vaccination	Immune from COVID-19	422 (40.04)
	Supporting the vaccination policies	507 (48.10)
	Requirement of workplace	125 (11.86)
Manufacturer of vaccines	Tianjin Cansino Biotechnology Inc.	25 (3.78)
	Beijing Institute of Biological Products Co., Ltd.	39 (5.89)
	Wuhan Institute of Biological Products and Wuhan Institute of Virology	103 (15.56)
	Sinovac Biotech Co., Ltd.	335 (50.60)
	Anhui Zhifei Longcom Biopharmaceutical Co., Ltd.	83 (12.54)
	Other manufacturers	11 (1.66)
	No knowledge of the manufacturer	66 (9.97)
The primary vaccination progress	Completed partial injections	530 (80.06)
	Completed all injections	132 (19.94)
Organizer of vaccination	Community	281 (42.45)
	School	176 (26.59)
	Workplace	189 (28.55)
	Other organizers	16 (2.42)
Source of vaccine information	Reports from the official online media	637 (34.98)
	Authoritative News media	522 (28.67)
	Social media	382 (20.98)
	Short video applications	118 (6.48)
	Recommendation of web pages	55 (3.02)
	Online health community	93 (5.11)
	Other sources	14 (0.77)

**Table 3:** Characteristics of Unvaccinated Participants (*n* = 124).

Variables		Participants, *n* (%)
Reasons for non-vaccination	Fear of side effects or adverse reactions of vaccines	37 (20.11)
	Doubts about the effectiveness of vaccines	14 (7.61)
	COVID-19 virus mutates too fast that vaccination may not prevent infection	19 (10.33)
	Research and development work of COVID-19 vaccine is immature yet	13 (7.07)
	Belief of the safety of living area and unnecessity of vaccination	26 (14.13)
	Personal health status	44 (23.91)
	In the process of getting other vaccines	10 (5.43)
Degree of vaccine hesitation (Regardless of the price of vaccine)	Very high	16 (12.90)
	High	18 (14.52)
	Fair	49 (39.52)
	Low	20 (16.13)
	Very low	21 (16.94)
Preference of the type of vaccine (Classified by the times of infections)	1 injection	36 (29.03)
	2 injections	34 (27.42)
	3 injections	10 (8.06)
	No preference for vaccine	44 (35.48)
Preference of the organizer of vaccination	Community	59 (47.58)
	School	25 (20.16)
	Workplace	39 (31.45)
	Other organizers	1 (0.81)
Source of vaccine information	Reports from the official online media	91 (32.27)
	Authoritative News media	81 (28.72)
	Social media	69 (24.47)
	Short video applications	15 (5.32)
	Recommendation of web pages	11 (3.90)
	Online health community	14 (4.96)
	Other sources	1 (0.35)

**Table 4:** Influencing factors of vaccine uptake between the vaccinated group and unvaccinated group.

Variables	Unvaccinated, *n* (%)	Vaccinated, *n* (%)	χ^2^/t	*p* Value
Gender			0.292	0.589
Male	52 (14.99)	295(85.01)		
Female	72 (16.40)	367(83.60)		
Age group			0.292	0.000
Under 18	15 (71.43)	6 (28.57)		
18–35	87 (14.10)	530 (85.90)		
36–59	14 (12.17)	101 (87.83)		
60 and above	8 (24.24)	25 (75.76)		
Living area			1.317	0.251
Urban	111 (16.37)	567 (83.63)		
Rural	13 (12.04)	95 (87.96)		
Highest level of education			7.216	0.065
High school and below	26 (24.30)	81 (75.70)		
Associate college	13 (13.98)	80 (86.02)		
College degree	50 (13.74)	314 (86.26)		
Master’s degree and above	35 (15.77)	187 (84.23)		
Occupation			14.660	0.041
Employees of enterprises and institutions	54 (20.15)	214 (79.85)		
Medical workers	2 (3.33)	58 (96.67)		
Civil servants	12 (11.88)	89 (88.12)		
Teachers	6 (12.24)	43 (87.76)		
Students	35 (15.98)	184 (84.02)		
Farmers	2 (8.33)	22 (91.67)		
Retired	9 (21.95)	32 (78.05)		
Others	4 (16.67)	20 (83.33)		
Non-vaccination due to personal health status			21.771	0.000
Yes	50 (40.32)			
No	74 (59.68)			
Attitudes towards the COVID-19 pandemic ^1^	2.93 ± 0.53	2.93 ± 0.59	−0.103	0.918
Attitudes towards the ^1^ COVID-19 vaccine	4.12 ± 0.70	4.39 ± 0.59	4.031	0.000
Exposure of COVID-19 vaccine Information			3.022	0.003
Very much	43 (34.68)	289 (43.66)		
Much	58 (46.77)	297 (44.86)		
Fair	17 (13.71)	72 (10.88)		
Less	5 (4.03)	4 (0.60)		
Very little	1 (0.81)	0 (0.00)		
Proportion of family members’ vaccine uptake			8.025	0.000
Very high	17 (4.56)	356 (95.44)		
High	51 (17.71)	237 (82.29)		
Fair	25 (37.31)	42 (62.69)		
Low	27 (51.92)	25 (48.08)		
Very low	4 (66.67)	2 (33.33)		
Proportion of close friends’ vaccine uptake			9.744	0.000
Very high	13 (5.96)	205 (94.04)		
High	66 (13.81)	412 (86.19)		
Fair	22 (33.85)	43 (66.15)		
Low	23 (92.00)	2 (8.00)		
Very low	0 (0.00)	0 (0.00)		

^1^ Quantitative data are described by mean ± standard deviation, and all other categorical variables are compared by independent sample *t* test.

**Table 5:** Results of binary logistic regression.

Characteristics	*β*	Standard Error	Wald	Degree of Freedom	*p* Value	Odds Ratio
Age group (Ref = 60 and above)			22.569	3	0.000	
Under 18	2.514	0.96	6.864	1	0.009	12.357
18–35	−0.258	0.758	0.116	1	0.733	0.773
36–59	−0.561	0.72	0.608	1	0.435	0.57
Occupation (Ref = Others)			9.112	7	0.245	
Employees of enterprises and institutions	0.215	0.655	0.108	1	0.742	1.24
Medical workers	−1.467	0.97	2.285	1	0.131	0.231
Civil servants	0.019	0.718	0.001	1	0.979	1.019
Teachers	0.113	0.791	0.02	1	0.887	1.119
Students	−0.211	0.677	0.097	1	0.755	0.81
Farmers	−1.475	1.134	1.691	1	0.193	0.229
Retired	0.135	0.945	0.021	1	0.886	1.145
Non-vaccination due to personal health status (Ref = No)	0.907	0.245	13.729	1	0.000	2.476
Proportion of family members’ vaccine uptake	−0.819	0.119	47.625	1	0.000	0.441
Proportion of close friends’ vaccine uptake	−0.896	0.184	23.595	1	0.000	0.408

## Data Availability

The data that support the findings of this study are available from the corresponding author upon reasonable request.
